# Genomic Analysis of *Staphylococcus aureus* of the Lineage CC130, Including *mecC*-Carrying MRSA and MSSA Isolates Recovered of Animal, Human, and Environmental Origins

**DOI:** 10.3389/fmicb.2021.655994

**Published:** 2021-03-25

**Authors:** Paula Gómez, Laura Ruiz-Ripa, Rosa Fernández-Fernández, Haythem Gharsa, Karim Ben Slama, Ursula Höfle, Myriam Zarazaga, Mark A. Holmes, Carmen Torres

**Affiliations:** ^1^Area of Biochemistry and Molecular Biology, OneHealth-UR Research Group, University of La Rioja, Logroño, Spain; ^2^Laboratoire des Microorganismes et Biomolécules Actives, Faculté des Sciences de Tunis, Université de Tunis El Manar, Tunis, Tunisia; ^3^Health and Biotechnology SaBio Research Group, Instituto de Investigación en Recursos Cinegéticos IREC (CSIC-UCLM-JCCM), Ciudad Real, Spain; ^4^Department of Veterinary Medicine, University of Cambridge, Cambridge, United Kingdom

**Keywords:** MRSA, MSSA, whole genome sequencing, CC130, ST700, IEC, *etD2*

## Abstract

Most methicillin resistant *Staphylococcus aureus* (MRSA) isolates harboring *mecC* gene belong to clonal complex CC130. This lineage has traditionally been regarded as animal-associated as it lacks the human specific immune evasion cluster (IEC), and has been recovered from a broad range of animal hosts. Nevertheless, sporadic *mecC*-MRSA human infections have been reported, with evidence of zoonotic transmission in some cases. The objective of this study was to investigate the whole-genome sequences of 18 *S. aureus* CC130 isolates [13 methicillin-resistant (*mecC*-MRSA) and five methicillin-susceptible (MSSA)] from different sequences types, obtained from a variety of host species and origins (human, livestock, wild birds and mammals, and water), and from different geographic locations, in order to identify characteristic markers and genomic features. Antibiotic resistance genes found among MRSA-CC130 were those associated with the SSC*mec*XI element. Most MRSA-CC130 strains carried a similar virulence gene profile. Additionally, six MRSA-CC130 possessed *scn-sak* and one MSSA-ST130 had *lukMF’*. The MSSA-ST700 strains were most divergent in their resistance and virulence genes. The pan-genome analysis showed that 29 genes were present solely in MRSA-CC130 (associated with SCC*mec*XI) and 21 among MSSA-CC130 isolates (associated with phages). The SCC*mec*XI, PBP3, GdpP, and AcrB were identical at the amino acid level in all strains, but some differences were found in PBP1, PBP2, PBP4, and YjbH proteins. An examination of the host markers showed that the 3’ region of the bacteriophage φ3 was nearly identical to the reference sequence. Truncated *hlb* gene was also found in *scn*-negative strains (two of them carrying *sak*-type gene). The *dtlB* gene of wild rabbit isolates included novel mutations. The *vwbp* gene was found in the three MSSA-ST700 strains from small ruminants and in one MSSA-ST130 from a red deer; these strains also carried a *scn*-type gene, different from the human and equine variants. Finally, a phylogenetic analysis showed that the three MSSA-ST700 strains and the two MSSA-ST130 strains cluster separately from the remaining MRSA-CC130 strains with the *etD2* gene as marker for the main lineage. The presence of the human IEC cluster in some *mecC*-MRSA-CC130 strains suggests that these isolates may have had a human origin.

## Introduction

*Staphylococcus aureus* is a common colonizer of the nasopharynx and skin of animals and humans; however, it is also a versatile opportunistic pathogen causing a wide variety of diseases from mild skin problems to life-threatening bacteraemias. The situation may be complicated when infections are caused by methicillin-resistant *S*. *aureus* (MRSA) isolates. Currently, the expression of *mecA* gene, as well as of other *mec* homolog genes, *mecC* and *mecB*, have been described in *S. aureus* conferring methicillin resistance (Becker et al., 2018).

The *mecC*-gene has been found in several MRSA lineages, mainly associated with animals, such as CC130, CC49, ST425, CC599, and CC1943. The ruminant associated CC130 is the most commonly found *me*cC lineage (Paterson et al., 2014a; Zarazaga et al., 2018). *mecC*-MRSA-CC130 was first described in cattle and in humans in the United Kingdom, Denmark, and Ireland (García-Álvarez et al., 2011; Shore et al., 2011). Since then, this lineage has been detected in diverse hosts in many European countries, with cattle and wildlife (including free grazing domesticated animals) being the most common hosts (Zarazaga et al., 2018). The prevalence of *mecC*-MRSA in people seems to be low (Paterson et al., 2014a, b; Lozano et al., 2020), however, the zoonotic transmission from livestock to people has been reported (Harrison et al., 2013), as well as its ability to cause disease (Petersen et al., 2013). This *mecC*-MRSA-CC130 lineage seems to be susceptible to many non-β-lactam agents and lacks major human virulence factors (Cuny et al., 2011; Monecke et al., 2013; Paterson et al., 2014a). However, they are carriers of a novel allele of exfoliative toxin gene (named *etd2*), which could explain the wide variety of hosts (Monecke et al., 2013). Adaptation of *S. aureus* to particular host species can be associated with mobile genetic elements (MGEs) or chromosomal mutations. In particular, the genes of the human specific immune evasion cluster (IEC) are considered to be a marker indicating some degree of human host adaptation. This IEC system is found in seven different configurations (types A–G) depending on the combination of five genes (*scn*, *chp*, *sak*, *sea*/*sep*); the *scn* gene (encodes a staphylococcal complement inhibitor) is included in all IEC types, and is often used as a marker of IEC-positive isolates, and is functionally essential (van Wamel et al., 2006). None of the *mecC*-MRSA reported strains harbored the *scn* gene (essential for the IEC system) (Lozano et al., 2020), with the exception of a few isolates belonging to ST1945, ST1581, and ST1583 previously described by our group from wildlife and extensively farmed domestic animals (Gómez et al., 2014, 2015; Ruiz-Ripa et al., 2019) and one ST1945 MRSA strain from a human sample (Harrison et al., 2017); it is worth noting that all these IEC-positive isolates were of type-E (carrying the *scn* and *sak* genes).

The methicillin-susceptible *S. aureus* (MSSA) isolates of the CC130 clonal complex are commonly found in cattle and are an important cause of disease (Monecke et al., 2016). The *mecA* gene has never been found in isolates belonging to the CC130 clonal complex and *S. aureus*-CC130 was initially described as a MSSA of animals from Europe and Africa (Smith et al., 2014). The ST700 lineage is part of CC130 by definition, as a single locus variant of ST130 (*tsi* allele different between them). MSSA-ST700 isolates are frequently found in Italian sheep populations (Azara et al., 2017; Vitale et al., 2018) and ST700 and some of its single locus variants (CC700) may be considered as a distinct, or separate, lineage due to its independent evolution and different epidemiology (Smith et al., 2014).

Studies of the intrinsic Penicillin-Binding-Proteins (PBPs) of *S. aureus* have shown that PBPs may contain mutations that affect β-lactam resistance, as highlighted by the case of a PBP4 capable of conferring high-level and broad-spectrum resistance to β-lactams, comparable to that provided by PBP2a (Chan et al., 2016).

In order to better understand the genetic characteristics of *S. aureus* CC130, the objective of this study was to analyze data from whole genome sequencing (WGS) of a collection of CC130 *S. aureus* strains (MRSA and MSSA) belonging to different sequences types, obtained from various host species, and from different geographic locations, in order to identify distinctive markers and genomic features of public health relevance.

## Materials and Methods

### Strains Included in the Study

Eighteen *S*. *aureus* strains of the clonal complex CC130 were included in this study for genomic comparison. These strains were as follows: (1) 13 MRSA, carrying the *mecC* gene, and belonging to the sequence types ST130, ST1945, ST3061, ST1571, ST1581, and ST1583; (2) two MSSA-ST130; and (3) three MSSA-ST700 (as a possible divergent CC130 lineage). These 18 MRSA-CC130, MSSA-ST130, and MSSA-ST700 strains were studied by WGS, having been collected during previous studies from different host samples: animals from extensive farms [four red deer (*Cervus elaphus*), two sheep (*Ovis* sp.), and one goat (*Capra* sp.)] (Gharsa et al., 2015; Gómez et al., 2015), wildlife [four magpies (*Pica pica*), two wild rabbits (*Oryctolagus cuniculus*), one wood mouse (*Apodemus sylvaticus*), one white stork (*Ciconia ciconia*), and one cinereous vulture (*Aegypius monachus*)] (Gómez et al., 2014, 2016; Ruiz-Ripa et al., 2019), the environment (one river water) (Gómez et al., 2017), and humans (one skin lesion of a cattle farmer)] (Benito et al., 2016). The characteristics of the included strains are indicated in Table 1.

**TABLE 1:** Characteristics of the 18 *S. aureus* CC130 strains (13 MRSA and 5 MSSA).

			Molecular typing		
Strain	Origin	Location (Region, country)	*spa*-type	Sequence-type (*arcC*, *aroE*, *glpF*, *gmk*, *pta*, *tpi*, *yqiL*)	Resistance Phenotype
C3817	Goat	Tunisia	t773	ST700 (6, 57, 45, 2, 7, 95, 52)	–
C3608	Sheep	Tunisia	t773	ST700 (6, 57, 45, 2, 7, 95, 52)	Tetracycline
C3630	Sheep	Tunisia	t7579	ST700 (6, 57, 45, 2, 7, 95, 52)	Tetracycline
C5802	River water	La Rioja, Spain	t843	ST130 (6, 57, 45, 2, 7, 58, 52)	Penicillin
C6771	Red Deer	Aragón, Spain	t1535	ST130 (6, 57, 45, 2, 7, 58, 52)	–
C7705	Red Deer	Cádiz, Spain	t1535	ST1945 (6, 57, 45, 2, 215, 58, 52)	Methicillin
C6595	Wood Mouse	Cádiz Spain	t1535	ST1945 (6, 57, 45, 2, 215, 58, 52)	Methicillin
C7708	Red Deer	Cádiz, Spain	t1535	ST1945 (6, 57, 45, 2, 215, 58, 52)	Methicillin
C7246	Farmer	La Rioja, Spain	t843	ST1945 (6, 57, 45, 2, 215, 58, 52)	Methicillin
C7925	White stork	Ciudad Real, Spain	t843	ST3061 (6, 57, 393, 2, 215, 58, 52	Methicillin
C7697	Red Deer	Cádiz, Spain	t843	ST1945 (6, 57, 45, 2, 215, 58, 52)	Methicillin
C8664	Magpie	Ciudad Real, Spain	t843	ST1583 (6, 57, 45, 2, 215, 58, 476)	Methicillin
C8666	Magpie	Ciudad Real, Spain	t843	ST1583 (6, 57, 45, 2, 215, 58, 476)	Methicillin
C8667	Magpie	Ciudad Real, Spain	t843	ST1583 (6, 57, 45, 2, 215, 58, 476)	Methicillin
C8671	Magpie	Ciudad Real, Spain	t843	ST1581 (417, 57, 45, 2, 215, 58, 476)	Methicillin
C8699	Cinereous vulture	Madrid, Spain	t843	ST1571 (6, 548, 45, 2, 215, 58, 52)	Methicillin
C8483	Rabbit	Aragón, Spain	t843	ST130 (6, 57, 45, 2, 7, 58, 52)	Methicillin
C8500	Rabbit	Aragón, Spain	t843	ST130 (6, 57, 45, 2, 7, 58, 52)	Methicillin

### Whole Genome Sequencing and Analysis of Sequences

Genomic DNA from each isolate was extracted with MasterPure^TM^ DNA Purification Gram Positive (Cambio, United Kingdom). WGS was performed on an Illumina HiSeq 2000 using paired-end mode (100 bp). *De novo* assembly and initial annotation was carried out using bioinformatic tools at the Wellcome Trust Sanger Institute. Reordering of the contigs was performed by alignment against *S. aureus* LGA251 genome (GenBank accession number: NC_017349) using Mauve (Rissman et al., 2009). Predicted coding sequences were identified and annotated automatically using RAST (Aziz et al., 2008) and manually with Genious Prime (Biomatters, Auckland, New Zealand). The resistance and virulence genotypes as well as the presence of *rep* genes were studied using ResFinder, VirulenceFinder and PlasmidFinder, respectively^1^. *In silico* analysis of the presence of antimicrobial substances related genes was performed using some genome-mining tools as antiSMASH and BAGEL (de Jong et al., 2006; Blin et al., 2019). PHASTHER Search Tool was used to determine the presence of prophage sequences (Arndt et al., 2016). When the study required it, the sequences were compared using Clustal Omega^2^.

The pan-genome was analyzed to estimate the core genome and the accessory or variable genome using Roary (Page et al., 2015) and BLAST-Ring-Image-Generator (BRIG) was employed to obtain a visual comparison with *S. aureus* LGA251 genome as reference (GenBank accession number: NC_017349) (Alikhan et al., 2011). Phylogenetic trees were generated using Geneious Prime with default settings.

## Results and Discussion

### Whole Genome Sequencing Results

The genome data of the 13 MRSA-CC130, two MSSA-ST130 and three MSSA-ST700 strains analyzed in this study have been placed in the European nucleotide archive^3^, and general sequence data, with the accession numbers are shown in Supplementary Table 1.

### Antimicrobial and Heavy Metal Resistance and Virulence Genotype

The resistance genotype analysis showed that all MRSA-CC130 strains contained the *mecC* as well as the *blaZ*-SCC*mec*XI (β-lactamase), *arsB* (arsenite efflux pump), and *arsC* (arsenate reductase) genes, which are described as being part of SCC*mec*XI element (Shore et al., 2011). No other resistance genes were detected among MRSA-CC130 strains, which agrees with the fully susceptible phenotype for non-β-lactams previously found in these *mecC*-positive strains. Among MSSA strains, three out of the five showed resistance to at least one of the antimicrobial agents tested, one MSSA-ST130 strain for penicillin (with *bla*Z gene) and two MSSA-ST700 strains for tetracycline [with *tet*(K) gene] (Table 2).

**TABLE 2:** Antimicrobial resistance and virulence genes detected in the 18 *S. aureus* CC130 strains included in this study.

Strain	Resistance genotype (antimicrobials and heavy metals)	Virulence genotype and host adaptation markers^a^
C3817	No related genes	***lukMF’***, *lukED*, *hlgAB*, *hlgCB*, ***tst-*variant, *sec*, *sel***, *edinB*, *splA/B/E*, *aur*, ***vwbp*, *scn*-type**
C3608	*tet*(K)	***lukMF’***, *lukED*, *hlgAB*, *hlgCB*, ***tst-*variant, *sec*, *sel*,** *edinB*, *splA/B/E*, *aur*, ***vwbp*, *scn*-type**
C3630	*tet*(K)	***lukMF’***, *lukED*, *hlgAB*, *hlgCB*, ***tst-*variant, *sec*, *sel*,** *edinB*, *splA/B/E*, *aur*, ***vwbp*, *scn*-type**
C5802	*blaZ*	***lukMF’***, *lukED*, *etD2*, *hlgAB*, *hlgCB, edinB, splA/B*, *aur*
C6771	No related genes	*lukED*, *etD2*, *hlgAB*, *hlgCB, edinB, splA/B/E*, *aur*, ***vwbp*, *scn*-type, *sak*-type**
C7705	*blaZ-*SSC*mec*XI, *mecC*, *arsB*, *arsC*	***scn*, *sak***, *lukED*, *etD2*, *hlgAB*, *hlgCB, edinB, splA/B/E*, *aur*
C6595	*blaZ-*SSC*mec*XI, *mecC*, *arsB*, *arsC*	***scn*, *sak***, *lukED*, *etD2*, *hlgAB*, *hlgCB, edinB, splA/B/E*, *aur*
C7708	*blaZ-*SSC*mec*XI, *mecC*, *arsB*, *arsC*	***scn*, *sak***, *lukED*, *etD2*, *hlgAB*, *hlgCB, edinB, splA/B/E*, *aur*
C7246	*blaZ-*SSC*mec*XI, *mecC*, *arsB*, *arsC*	***sak***, *lukED*, *etD2*, *hlgAB*, *hlgCB, edinB, splA/B/E*, *aur*
C7925	*blaZ-*SSC*mec*XI, *mecC*, *arsB*, *arsC*	*lukED*, *etD2*, *hlgAB*, *hlgCB, edinB, splA/B/E*, *aur*
C7697	*blaZ-*SSC*mec*XI, *mecC*, *arsB*, *arsC*	***scn*, *sak***, *lukED*, *etD2*, *hlgAB*, *hlgCB, edinB, splA/B/E*, *aur*
C8664	*blaZ-*SSC*mec*XI, *mecC*, *arsB*, *arsC*	***scn*, *sak***, *lukED*, *etD2*, *hlgAB*, *hlgCB, edinB, splA/B/E*, *aur*
C8666	*blaZ-*SSC*mec*XI, *mecC*, *arsB*, *arsC*	*lukED*, *etD2*, *hlgAB*, *hlgCB, edinB,splA/B/E*, *aur*
C8667	*blaZ-*SSC*mec*XI, *mecC*, *arsB*, *arsC*	*lukED*, *etD2*, *hlgAB*, *hlgCB, edinB, splA/B/E*, *aur*
C8671	*blaZ-*SSC*mec*XI, *mecC*, *arsB*, *arsC*	***scn*, *sak***, *lukED*, *etD2*, *hlgAB*, *hlgCB, edinB, splA/B/E*, *aur*
C8699	*blaZ-*SSC*mec*XI, *mecC*, *arsB*, *arsC*	*lukED*, *etD2*, *hlgAB*, *hlgCB, edinB, splA/B/E*, *aur*, ***sak-*type**
C8483	*blaZ-*SSC*mec*XI, *mecC*, *arsB*, *arsC*	*lukED*, *etD2*, *hlgAB*, *hlgCB, edinB, splA/B*, *aur*
C8500	*blaZ-*SSC*mec*XI, *mecC*, *arsB*, *arsC*	*lukED*, *etD2*, *hlgAB*, *hlgCB, edinB, splA/B*, *aur*

*^a^In bold are highlighted the genes associated with: human immune evasion system, bovine leucocidin, toxic shock syndrome, enterotoxins, von Willebrand factor-binding protein, and staphylococcal complement inhibitor and staphylokinase-type genes (of about 45% of amino acid similarity).*

A list of selected virulence and/or fitness genes are shown in Table 2. All the strains carried the genes: *lukED*, *hlgAB*, *hlgCB*, *edinB*, *splA*/*B*, and *aur*. Nevertheless, some differences were detected with respect to genes belonging to the IEC system, leucocidins, exfoliative toxins, allele variant of toxic shock syndrome toxin, enterotoxins, and immune evasion proteases. The three MSSA strains of lineage ST700 carried *sec* and *sel* genes, and also a variant of *tst* with an amino acid sequence closer to the *tst* gene found associated with bovine origin than with the one of human origin (Monecke et al., 2007); this combination of pyrogenic toxin superantigens is associated with the pathogenicity island SaPIbov (Fitzgerald et al., 2001), and has been previously described in strains from ruminants with the same ST (Luzzago et al., 2014). The ST700 strains were obtained from apparently healthy animals although a subclinical mastitis cannot be ruled out. All of them presented the *tst-variant*, *sec*, and *sel* virulence genes, as well as the *lukMF*’ gene, previously found in isolates from cases of mastitis (Schlotter et al., 2012). All our CC130 strains, except those belonging to ST700, carried the *etD2* gene. The *lukMF*’ genes, encoding a leucocidin strongly associated with ruminants (Monecke et al., 2007), were only detected in four MSSA strains obtained from sheep and goats (MSSA-ST700) and from river water (MSSA-ST130); these data support the association of this leucocidin with a ruminant origin, and also may suggests that the strain from river water could have a bovine origin.

On the other hand, the *lukED*, *hlgAB*, *hlgCB*, *edinB*, *splA*/*B*, and *aur* genes were present in the 18 strains. Usually, *S. aureus* has up to 6 types of toxin genes in the core genome (HlgAB, HlgCB, and LukAB) (Alonzo and Torres, 2014). The combination of LukED with *splA*/*splB* genes has been detected previously among other clonal complexes (Jamrozy et al., 2012), generally being found on the genomic island νSaβ, highly conserved in some lineages (McCarthy and Lindsay, 2013). Other genes, such as *aur* (immune evasion proteases), *edinB* (exfoliative toxin) or *splA*/*B*/*E* (immune evasion proteases), are found highly conserved in *S. aureus* (Sabat et al., 2008; Munro et al., 2010; Paharik et al., 2016). Nevertheless, the *splE* gene was absent in three of our strains, and some authors suggested the implication of this nuclease in clinical manifestations (Stach et al., 2018). The analysis of genes encoding bacteriocins revealed the presence of the gene encoding the bacteriocin lactococcin 972 (GenBank accession number: NC_004955) in all the analyzed strains; furthermore, this gene showed in all isolates an identical genetic environment, which corresponds to the one found in the reference sequence of *S. aureus* LGA251.

### Comparison Between the Strains

The pan-genome study showed that a total of 2,318 genes were included in all strains, 539 were in two or more strains, and 345 were unique genes of specific strains. Circular genome comparison of MSSA and MRSA strains (LGA251 as reference) showed some differences between MRSA and MSSA strains (Supplementary Figure 1). It was determined that 29 genes were present in all 13 MRSA and in none of the MSSA strains (mainly associated with SCC*mec*XI mobile genetic element), and 21 genes in all 5 MSSA strains and not in the MRSA (mostly associated with phages) (Supplementary Table 2). It has been reported that the core genome is largely preserved within the same lineage (McCarthy et al., 2011). In addition, we analyzed the presence of unique genes in *scn*-positive (*n* = 6) and *scn*-negative strains (*n* = 12). The *scn*-negative strains did not carry unique genes, however, *scn*-positive strains presented different genes encoding proteins associated with bacteriophages (including the human *scn*-IEC gene), that were not present among *scn*-negative strains (Supplementary Table 3).

### SCC*mec*XI Element and Penicillin Binding Proteins (PBPs)

The structure of the SCC*mec*XI element in the 13 MRSA CC130 strains was compared with the one of MRSA strain M10/0061 (GenBank accession number: FR823292), used as reference. This structure seems to be highly conserved among the 13 MRSA-CC130 strains, pointing out the potential of this type of SCC*mec* to be transferred among S. *aureus*, due to the relatively small size of this mobile genetic element, approximately 30 Kb (Shore et al., 2011). In fact, it has been suggested that SCC*mec*XI could have originated in another species or genus, being distantly related to the other SCC*mec* elements and possibly SCC*mec*XI represent an ancestral form (Shore et al., 2011).

The results of the study of amino acid changes in PBPs and in other three proteins previously associated with β-lactam resistance (Ba et al., 2014, 2019) are shown in Table 3. Amino acid changes in our strains were included, as well as those of MRSA LGA251 using the corresponding sequences of MSSA ATCC 25923 (GenBank accession number: CP009361) as reference for all sequences, except for PBP2c. In the case of PBP2c, the sequence of MRSA LGA251 was used as reference. The PBP2c protein, encoded by the *mecC* gene, presented a 100% of amino acid similarity to PBP2c of MRSA LGA251. As regards the other PBPs, some amino acid changes were found, especially in PBP3 and PBP4. These amino acid changes seem to be repeated in the 18 strains, including MSSA ones, with some few exceptions (PBP1: T371I in a MSSA-ST130 water strain; PBP2: T439M in a MRSA-ST1583 magpie strain; PBP4: D28N in MSSA-ST700 strains, and A288T in two MRSA-ST130 rabbit strains). Nevertheless, differences were greater in the case of MRSA LGA251. It should be noted that previous studies indicate that mutations in PBP4 are related to increased MICs for β-lactams (Alexander et al., 2018), and a modified PBP1 had been previously associated with a reduced susceptibility in *S. lugdunensis*, but not in *S. aureus* (Kotsakis et al., 2012). Only one of the changes detected in this study, T371I in PBP1, was previously reported, combined in that case with other PBP mutations in a clinical MRSA ST1 strain lacking *mec* gene (Ba et al., 2014); in our case, the strain which harbored the T371I change was MSSA. In addition to PBPs, the study of GdpP, YjbH, and AcrB proteins, which could be implicated in β-Lactam resistance (Banerjee et al., 2010; Göhring et al., 2011; Ba et al., 2019), showed the same amino acid changes in all analyzed strains, with the exception of YjbH in which two changes (L95V, A83P) were detected in all strains, but in MSSA-ST700, MRSA-ST130 and MSSA-CC130 where only one change was found (L95V).

**TABLE 3:** Identified amino acid changes in PBPs 1, 2, 2c, 3, 4, YjbH, GdpP, and AcrB proteins of the 18 *S. aureus* strains included in this study and also of MRSA LGA251 strain (MRSA LGA251 as used as reference strain for PBP2c and MSSA ATCC 25923 as reference strain for PBPs 1, 2, 3, 4, YjbH, GdpP, and AcrB protein analysis).

Strain	ST	PBP1	PBP2	PBP2c	PBP3	PBP4	YjbH	GdpP	AcrB
LGA 251	ST425	Wild	T439V, T691A, A705V	Wild	M1L, K504R, D563E	E398A	L95V	I456V, D561E	S52T, L198V, T282A, E456D, T577A, S861F
C3817	ST700	Wild	T439V	–	M1L, K160N, K504R, D563E	D28N, K349E, E398A	L95V	I456V, D561E	S52T, T282A, T577A
C3608	ST700	Wild	T439V	–	M1L, K160N, K504R, D563E	D28N, K349E, E398A	L95V	I456V, D561E	S52T, T282A, T577A
C3630	ST700	Wild	T439V	–	M1L, K160N, K504R, D563E	D28N, K349E, E398A	L95V	I456V, D561E	S52T, T282A, T577A
C5802	ST130	T371I	T439V	–	M1L, K160N, K504R, D563E	K349E, E398A	L95V	I456V, D561E	S52T, T282A, T577A
C6771	ST130	Wild	T439V	–	M1L, K160N, K504R, D563E	K349E, E398A	L95V	I456V, D561E	S52T, T282A, T577A
C7705	ST1945	Wild	T439V	Wild	M1L, K160N, K504R, D563E	K349E, E398A	L95V, A83P	I456V, D561E	S52T, T282A, T577A
C6595	ST1945	Wild	T439V	Wild	M1L, K160N, K504R, D563E	K349E, E398A	L95V, A83P	I456V, D561E	S52T, T282A, T577A
C7708	ST1945	Wild	T439V	Wild	M1L, K160N, K504R, D563E	K349E, E398A	L95V, A83P	I456V, D561E	S52T, T282A, T577A
C7246	ST1945	Wild	T439V	Wild	M1L, K160N, K504R, D563E	K349E, E398A	L95V, A83P	I456V, D561E	S52T, T282A, T577A
C7925	ST3061	Wild	T439V	Wild	M1L, K160N, K504R, D563E	K349E, E398A	L95V, A83P	I456V, D561E	S52T, T282A, T577A
C7697	ST1945	Wild	T439V	Wild	M1L, K160N, K504R, D563E	K349E, E398A	L95V, A83P	I456V, D561E	S52T, T282A, T577A
C8664	ST1583	Wild	T439V	Wild	M1L, K160N, K504R, D563E	K349E, E398A	L95V, A83P	I456V, D561E	S52T, T282A, T577A
C8666	ST1583	Wild	T439V	Wild	M1L, K160N, K504R, D563E	K349E, E398A	L95V, A83P	I456V, D561E	S52T, T282A, T577A
C8667	ST1583	Wild	T439M	Wild	M1L, K160N, K504R, D563E	K349E, E398A	L95V, A83P	I456V, D561E	S52T, T282A, T577A
C8671	ST1581	Wild	T439V	Wild	M1L, K160N, K504R, D563E	K349E, E398A	L95V, A83P	I456V, D561E	S52T, T282A, T577A
C8699	ST1571	Wild	T439V	Wild	M1L, K160N, K504R, D563E	K349E, E398A	L95V, A83P	I456V, D561E	S52T, T282A, T577A
C8483	ST130	Wild	T439V	Wild	M1L, K160N, K504R, D563E	A288T, K349E, E398A	L95V	I456V, D561E	S52T, T282A, T577A
C8500	ST130	Wild	T439V	Wild	M1L, K160N, K504R, D563E	A288T, K349E, E398A	L95V	I456V, D561E	S52T, T282A, T577A

### Host Adaptation, Prophages, and Other Mobile Genetic Elements

**FIGURE 1:**
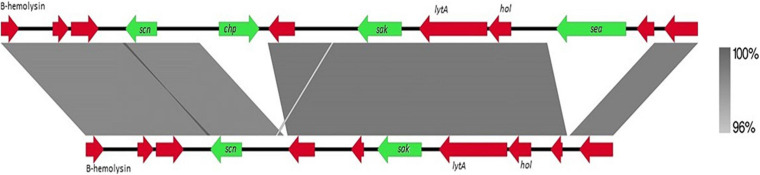
Comparison of 3′ conserved region of β-hemolytic bacteriophage (Φ3) between reference strain MRSA-252 (above) and C6595 (below). The percentage of similarity is indicated (right). Arrows in green corresponding to the IEC genes, and in red other coding sequences.

The presence of the *scn* gene in some of our *mec*C-positive MRSA-CC130 strains is a remarkable feature since *mecC*-MRSA, as well as CC130 strains in general, are considered animal-associated, and IEC system is considered a human adaptation marker. The 3’ conserved region of the β-hemolytic bacteriophage φ3 (approximately 8,000 pb) of the *mecC*-positive strain C6595 (IEC type E, isolated of a wood mice) was compared with the IEC of the reference strain MRSA252 (GenBank accession number: BX571856, type A) (Figure 1), and no differences were observed apart from the different content in genes that give rise to the type of IEC. It can be assumed that these strains have an advantage in colonizing and/or infecting humans, as already was described in unusual IEC-positive MRSA livestock associated CC398 strains (Cuny et al., 2015; Pérez-Moreno et al., 2017; Ceballos et al., 2019).

The *hlb* gene was also analyzed, showing that it was truncated in all the strains that presented the IEC system. The truncated *hlb* gene was also found in other three *scn*-negative strains (C6771, C7246, and C8699), which showed an integrated phage of about 45 kb, that only contained phage-related genes; it should be highlighted that C6771 and C8699 isolates contained a *sak*-related gene with a 45% of similarity respect to the *sak* IEC virulence gene (GenBank accession number: NC_026016). The *dtlB* gene, present in the two MRSA-ST130 *mecC*-positive strains isolated from healthy wild rabbits (C8483 and C8500), showed the following amino acid changes with respect to the reference MRSA252 strain (GenBank accession number: BX571856): (a) C8483 (I227T, A382S, and ^∗^405Q); (b) C8500 (A382S, G401D, and K402R). None of the strains presented the mutations T113K, Y250H, or ^∗^405Y previously described (Viana et al., 2015), and only the A382S change present in both strains has not been previously reported (Viana et al., 2015; Holmes et al., 2016). The *vwbp* gene (SaPIbov5, GenBank accession number: JP5338 used as reference) was found in the three MSSA-ST700 strains from small ruminants and in the MSSA-ST130 strain from a red deer, indicating in this case an adaptation to the host (Viana et al., 2010).

Phage analysis showed 12 different intact prophages in genome with an average of 2 ± 2 prophage regions per genome. The strains C3608, C3630, C3817, and C6771 (3 MSSA-ST700 and one MSSA-ST130, that also carried the *vwbp* gene), showed an identical coding sequence contained in a phage described as a protein related to the expression of fibrinogen (*scn*-type gene), but different from human variant (47% similarity of amino acid sequence with WP_000702262 as reference) and from the new variant described and associated with the evasion of the equine immune system (45% similarity of amino acid sequence with WP_106096712 as reference) (Supplementary Figure 2).

Only three strains (C3608, C3630 and C5802, corresponding with two MSSA-ST700 and one MSSA-ST130), presented *rep* genes: *rep*_7_, *rep*_24_, *rep_*US*23_*, *rep*_5_. In addition, only *rep*_7_ was detected showing a 100% nucleotide identity in two of the strains (C3608 and C3630). The gene *rep*_7_ has been previously described widely distributed in other CCs within the species *S. aureus* (Lozano et al., 2012).

### Phylogenetic Analysis

**FIGURE 2:**
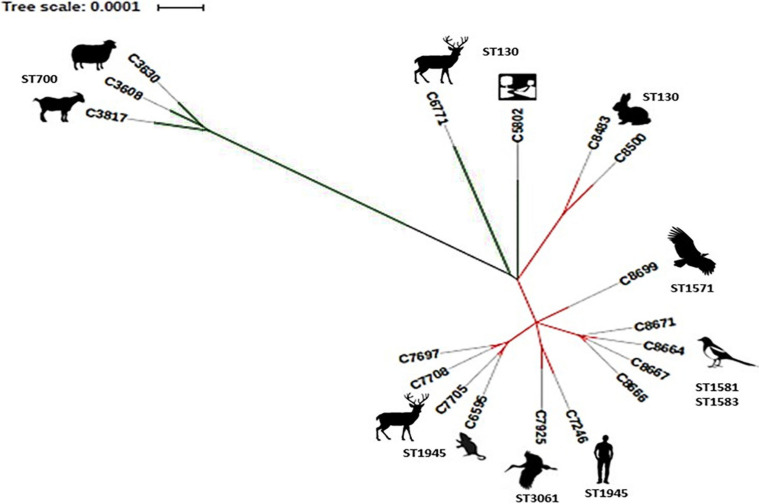
Phylogenetic tree showing the phylogenetic distance between 18 strains CC130 included in this study. Green lines corresponding to MSSA and red lines to MRSA strains.

The phylogenetic tree (Figure 2) showed that the three MSSA strains belonging to ST700, clearly constituted a separate clade from the remaining CC130 strains included in this study. Our ST700 MSSA strains were very different from the other 15 strains of the studied collection based on the results from all the analyses performed, supporting the consideration of ST700 as a lineage distinct from CC130 (Smith et al., 2014). The other 2 MSSA-ST130 strains also form a distinct clade in this collection.

Finally, the 13 MRSA-CC130 strains are grouped and the following associations can be seen: (1) C8483 and C8500 from rabbits from Aragon are clustered together indicating the possible animal-animal transfer; (2) C6595, C7697, C7705, and C7708, from red deer and small mammals from the same geographical area, highlighting C7705 and C6595 MRSA strains that were indistinguishable (by this analysis), which might suggest an interspecies transmission event; (3) C8666, C8667, C8664, and C8671, all isolated from magpies in the same location; (4) C7925 and C7246 that were isolated in a different geographical area, of different origins (stork and human, respectively), and with different STs; and (5) C8699 (from vulture) that is grouped individually.

## Conclusion

Taking into account the relatively small number of strains included in this study, the comparison of fifteen strains CC130 from different animal origins, geographical locations and STs, demonstrated clear differences between isolates depending if they were *mecC*-positive or *mecC*-negative and between sequence types. Markedly divergent results from the three MSSA-ST700 isolates reinforce the idea of considering this lineage as distinctly separate from CC130. The *etD2* gene appears to be a genetic marker of CC130 lineage (MSSA and MRSA), which is missing from ST700 strains although further studies are required to confirm this. The presence of IEC system in some of the MRSA-*mecC* from animals opens questions about the origins and evolution of *mecC*-MRSA.

## Data Availability Statement

The datasets presented in this study can be found in online repositories. The names of the repository/repositories and accession number(s) can be found in the article/Supplementary Material.

## Author Contributions

CT and MH conceived and designed the study. PG, LR-R, RF-F, HG, KB, and UH performed the initial sampling procedure and the initial characterization of isolates. PG performed laboratory work. PG, MZ, CT, and MH interpreted the results and contributed to producing the first draft of the manuscript. All authors have revised and agreed to the final version of the manuscript.

## Conflict of Interest

The authors declare that the research was conducted in the absence of any commercial or financial relationships that could be construed as a potential conflict of interest.

## Footnotes

1 http://www.genomicepidemiology.org/

2 https://www.ebi.ac.uk/tools

3 http://www.ebi.ac.uk/ena

## Supplementary Material

Supplementary Figure 1 Circular comparison of MSSA in Green-blue rings and MRSA in warm colors using LGA251 as reference.% GC content and GC Skew are represented in innermost circles (colors indicated in the coded legend).

Supplementary Figure 2 **(A)** Amino acid comparative of *scn*-like found in 4 MSSA isolates of this study. **(B)** Amino acid comparative between one *scn*-like found in this study, one *scn*-equine (WP_106096712 as reference) and one *scn*-human (WP_000702262 as reference).

Supplementary Table 1 Results of whole genome sequence and accession number of the strains included in this study.

Supplementary Table 2 Result of unique genes in MRSA and in MSSA strains analyzed by Roary pipeline.

Supplementary Table 3 Result of unique genes in *scn* positive and *scn* negative strains analyzed by Roary pipeline.
