# Supplementary material for: Genomic Analysis of Staphylococcus aureus of the Lineage CC130, Including mecC-Carrying MRSA and MSSA Isolates Recovered of Animal, Human, and Environmental Origins
Source: Front Microbiol. 2021 Mar 25;12:655994. doi: 10.3389/fmicb.2021.655994 (PMC8027229; doi:10.3389/fmicb.2021.655994)
Supplement: Supplementary Figure 1 — Circular comparison of MSSA in Green-blue rings and MRSA in warm colors using LGA251 as reference.% GC content and GC Skew are represented in innermost circles (colors indicated in the coded legend). [file Data_Sheet_2.docx]

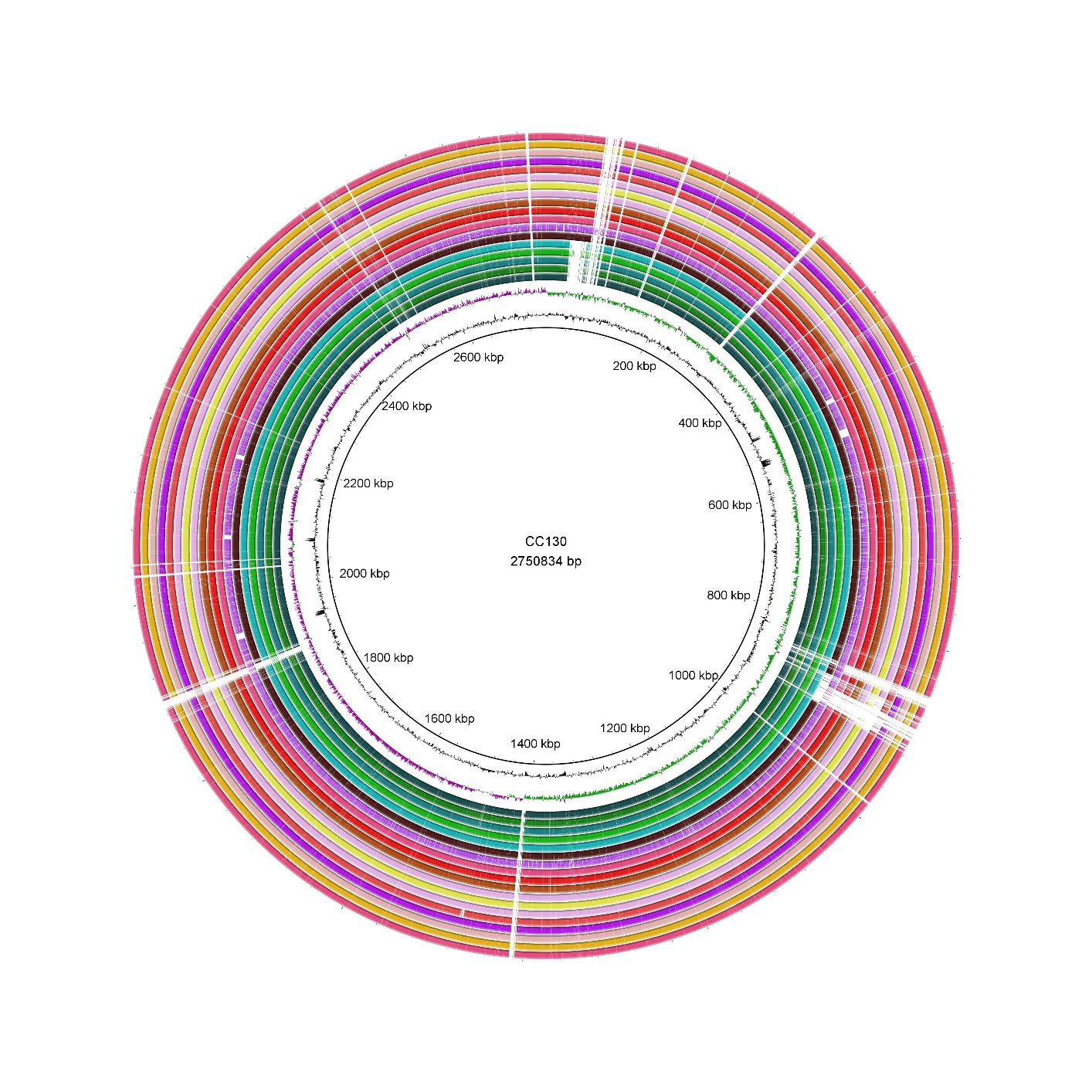


**Supplementary figure 1**. Circular comparison of MSSA in Green-blue rings and MRSA in warm colours using LGA251 as reference. % GC content and GC Skew are represented in innermost circles (colours indicated in the coded legend).

**a)**

**C3608** MKLKKCIMTTALAIGVAASSIGVYEGSAKASTDKQITANEYYDQKLAKELKDLLNELNVNVLATSSLDPYYKRNVQ

**C3817** MKLKKCIMTTALAIGVAASSIGVYEGSAKASTDKQITANEYYDQKLAKELKDLLNELNVNVLATSSLDPYYKRNVQ

**C3630** MKLKKCIMTTALAIGVAASSIGVYEGSAKASTDKQITANEYYDQKLAKELKDLLNELNVNVLATSSLDPYYKRNVQ

**C6771** MKLKKCIMTTALAIGVAASSIGVYEGSAKASTDKQITANEYYDQKLAKELKDLLNELNVNVLATSSLDPYYKRNVQ

**C3608** MYGFKAKMVVKSKNYSKMSIAKEELENIYREIDEALANYY

**C3817** MYGFKAKMVVKSKNYSKMSIAKEELENIYREIDEALANYY

**C3630** MYGFKAKMVVKSKNYSKMSIAKEELENIYREIDEALANYY

**C6771** MYGFKAKMVVKSKNYSKMSIAKEELENIYREIDEALANYY

**b)**

**C3608** MKLKKCIMTTALAIGVAASSIGVYEGSAKASTDKQITANEYYDQKLAKELKDLLNELNVN

**human** MKIRKSILAGTLAIVLASPLVTNLDKNEAQASTSLPTSNEYQNEKLANELKSLLDELNVN

**equine** MRGKKHIIAGVLVAVLSTPLVTSFESKEVKAES—INANTYINQNLEKELRELLDELNVN

MKLKKCIITTTLAIGVTVSSIGLHEGTAKASTYKQITANEYYDQKLAKELKGLLDELNVN

**C3608** VLATSSLDPYYKRNVQMYGFKAKMVVKSKNYSKMSIAKEELENIYREIDEALANYY

**human** ELATGSLNTYYKRTIKISGLKAMYALKSKDFKKMSEAKYQLQKIYNEIDEALKSKY

**equine** ELATGSLNPYFKRTVKKYGYKAKAALRSKDFTRMSQSKYELQSIYSEIDKALGYER

VLATGSLDPYYKRNVLMYGFKAKMALKSKNYSKMSIAKEELENFYREIDEALANDY

**Supplementary figure 2**. a) Amino acid comparative of *scn*-like found in 4 MSSA isolates of this study. b) Amino acid comparative between one *scn*-like found in this study, one *scn*-equine (WP_106096712 as reference) and one *scn*-human (WP_000702262 as reference).
