# Supplementary material for: Genomic Analysis of Staphylococcus aureus of the Lineage CC130, Including mecC-Carrying MRSA and MSSA Isolates Recovered of Animal, Human, and Environmental Origins
Source: Front Microbiol. 2021 Mar 25;12:655994. doi: 10.3389/fmicb.2021.655994 (PMC8027229; doi:10.3389/fmicb.2021.655994)
Supplement: Supplementary Table 1 — Results of whole genome sequence and accession number of the strains included in this study. [file Data_Sheet_1.docx]

**Supplementary Table 1.** Results of whole genome sequence analysis and accession numbers of the strains included in this study.

| **Strain** | **Accession number (ENA)** | **Genome size (pb)** | **Number of contigs** |
| --- | --- | --- | --- |
| C3608 | ERS659506 | 2853412 | 28 |
| C3630 | ERS659507 | 2815858 | 27 |
| C3817 | ERS659508 | 2809780 | 23 |
| C5802 | ERS659514 | 2879014 | 23 |
| C6595 | ERS659521 | 2778052 | 14 |
| C6771 | ERS1026449 | 2779822 | 20 |
| C7246 | ERS659535 | 2774525 | 19 |
| C7697 | ERS659537 | 2778002 | 15 |
| C7705 | ERS659538 | 2779369 | 17 |
| C7708 | ERS659539 | 2778545 | 17 |
| C7925 | ERS659543 | 2694187 | 16 |
| C8483 | ERS1026426 | 2753568 | 15 |
| C8500 | ERS1026369 | 2761300 | 17 |
| C8664 | ERS1026371 | 2809520 | 23 |
| C8666 | ERS1026373 | 2769845 | 27 |
| C8667 | ERS1026375 | 2770018 | 30 |
| C8699 | ERS1026424 | 2749249 | 15 |
| C8671 | ERS1026380 | 2809991 | 25 |

**Supplementary Table 2.** Result of unique genes among MRSA-CC130 and MSSA-CC130 strains analysed by Roary pipeline.

| MRSA (n=13) | FAD-binding dehydrogenase | Flavin reductase like domain protein | *ccrB*/cassette chromosome recombinase B | *mecR1*/methicillin resistance protein MecR1 |
| --- | --- | --- | --- | --- |
|  | *lolD*/ABC-type transport system ATPase | ABC transporter permease | Pyridoxal phosphate-dependent enzyme | *mecC*/beta-lactam-inducible penicillin-binding protein |
|  | *arsR*/arsenical resistance operon repressor | *lip*/triacylglycerol lipase | Truncated DNA repair protein RadC | *blaZ*/beta-lactamase |
|  | *arsB*/arsenical pump membrane protein | *dinG*/DnaQ family exonuclease/DinG family helicase | Putative DNA binding protein | 8 hypothetical proteins |
|  | *arsC*/arsenate reductase | Putative ATP-binding protein | Telomeric repeat-binding factor 2 |  |
|  | Major facilitator superfamily protein | *ccrA1*/site-specific recombinase CcrA1 | *mecI*/methicillin resistance regulatory protein MecI |  |
| MSSA (n=5) | Superfamily II helicase | Putative phi PVL-like protein | Phage major capsid protein, HK97 family | 9 phage proteins |
|  | Phage terminase, large subunit, PBSX family | Putative head-tail adaptor | Phage gp6-like head-tail connector family protein |  |
|  | Phage minor head protein | Phage tape measure protein | Putative tail protein |  |
|  | Putative phage PVL protein | Phage portal protein, SPP1 family | Phage tail family protein |  |

**Supplementary Table 3.** Result of unique genes in *scn*-positive and *scn*-negative *S. aureus* CC130 strains analysed by Roary pipeline.

| *scn* positive  (n=6) | Amidase | Putative cytosolic protein | Phage DNA-binding protein |
| --- | --- | --- | --- |
|  | Putative phi ETA-like protein | HIRAN domain-containing protein | Prophage L54a, antirepressor |
|  | Putative phi PVL-like protein | Repressor-like protein | 3 phage proteins |
|  | *scn*/Involved in expression of fibrinogen binding protein, phage associated |  |  |
| *scn* negative  (n=12) | - | | |
